# Evaluating the Performance of Deep Learning Frameworks for Malaria Parasite Detection Using Microscopic Images of Peripheral Blood Smears

**DOI:** 10.3390/diagnostics12112702

**Published:** 2022-11-05

**Authors:** Dilber Uzun Ozsahin, Mubarak Taiwo Mustapha, Basil Bartholomew Duwa, Ilker Ozsahin

**Affiliations:** 1 Department of Medical Diagnostic Imaging, College of Health Science, University of Sharjah, Sharjah 27272, United Arab Emirates; 2 Operational Research Centre in Healthcare, Near East University, TRNC Mersin 10, Nicosia 99138, Turkey; 3 Department of Biomedical Engineering, Near East University, TRNC Mersin 10, Nicosia 99138, Turkey; 4 Brain Health Imaging Institute, Department of Radiology, Weill Cornell Medicine, New York, NY 10065, USA

**Keywords:** blood smear, detection, malaria, parasite, transfer learning

## Abstract

Malaria is a significant health concern in many third-world countries, especially for pregnant women and young children. It accounted for about 229 million cases and 600,000 mortality globally in 2019. Hence, rapid and accurate detection is vital. This study is focused on achieving three goals. The first is to develop a deep learning framework capable of automating and accurately classifying malaria parasites using microscopic images of thin and thick peripheral blood smears. The second is to report which of the two peripheral blood smears is the most appropriate for use in accurately detecting malaria parasites in peripheral blood smears. Finally, we evaluate the performance of our proposed model with commonly used transfer learning models. We proposed a convolutional neural network capable of accurately predicting the presence of malaria parasites using microscopic images of thin and thick peripheral blood smears. Model evaluation was carried out using commonly used evaluation metrics, and the outcome proved satisfactory. The proposed model performed better when thick peripheral smears were used with accuracy, precision, and sensitivity of 96.97%, 97.00%, and 97.00%. Identifying the most appropriate peripheral blood smear is vital for improved accuracy, rapid smear preparation, and rapid diagnosis of patients, especially in regions where malaria is endemic.

## 1. Introduction

Malaria is an infection of the red blood cells caused by the protozoan parasites that belong to the genus Plasmodium [1]. These parasites are mainly passed on to humans by the bites of female Anopheles mosquitoes that have been infected [2]. Thus, it is an infectious disease that is spread not only to people but also to animals. Malaria can cause various symptoms, the most common of which are fever, exhaustion, nausea, and headaches [3]. In extreme circumstances, it can induce jaundice, seizures, coma, and even death [4]. Symptoms typically appear 10–15 days following a mosquito bite [3]. If the condition is not effectively managed, patients may experience relapses months later [3]. In those who have recently recovered from an infection, reinfection typically results in milder symptoms [5]. However, partial resistance diminishes over months to years if the individual is not continually exposed to malaria [6]. There are approximately 156 named species of Plasmodium that infect various human species of vertebrates [2]. However, only four species are considered true parasites of humans. Malaria is caused by six species of Plasmodium which infect humans [7]. They include Plasmodium falciparum (*P. falciparum*), Plasmodium malaria (*P. malariae*), Plasmodium ovale curtisi (*P. ovale curtisi*), Plasmodium ovale wallikeri (*P. ovale wallikeri*), Plasmodium vivax (*P. vivax*), and Plasmodium knowlesi (*P. knowlesi*). Approximately 75% of infected people have P. falciparum, followed by 20% who have *P. vivax* [7].

**Figure 1:**
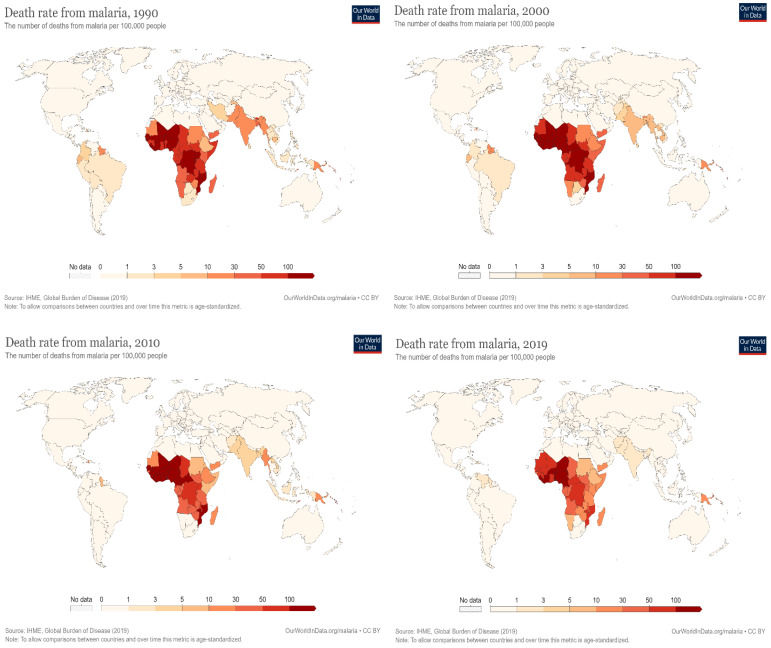
The global mortality rate of malaria “Adapted with permission from Ref. [14]”.

A survey by the Centers for Disease Control and Prevention (CDC) in 2020 recorded an estimated global mortality rate of more than 600,000 deaths from malaria, with a higher percentage from Sub-Saharan Africa, the majority of whom were children and pregnant women [8]. In a similar report by the World Health Organization (WHO), there were around 409,000 fatalities and 229 million new malaria cases in 2019 [9]. A total of 67% of all malaria deaths in 2019 occurred in children under five [10]. The WHO also projected about 3.3 million malaria cases to occur globally each year [10]. Furthermore, around 125 million pregnant women worldwide risk infection each year. It is estimated that maternal malaria causes up to 200,000 baby deaths annually in Sub-Saharan Africa alone. In Western Europe, there are approximately 10,000 cases of malaria per year, whereas in the United States, there are about 1300–1500 cases [11]. Although there are occasional cases, the United States eliminated malaria as a serious threat to public health in 1951 [12]. In Europe, the disease was responsible for the deaths of around 900 persons between the years 1993 and 2003 [13]. In recent years, there has been a global downward trend in the incidence of disease and the mortality rate that it causes, as shown in Figure 1 [14].

Artificial intelligence in medical diagnosis has gained much popularity in the last few decades [15,16,17]. Previous studies have indicated the possibility of implementing deep learning frameworks in malaria parasite detection. Maqsood et al. [7] studied microscopic thin blood smear images to detect malaria parasites.

This process is required due to the public health quest for an immediate diagnostic remedy to various diseases using deep learning approaches. Their study proposed an advanced customized convolutional neural network (CNN) model to outperform other contemporary deep learning models. Therefore, the image augmentation method and the bilateral filtering were featured in the red blood cells before model training. The dataset was retrieved from the NIH Malaria dataset, which revealed the proposed experimental result algorithm to be 96.82%, perfect for malaria detection. However, their study only focuses on the thin blood smear of the Plasmodium images [7]. Similarly, Yang et al. [8] proposed a deep-learning study for smartphone-based Plasmodium detection of malaria parasites using thick blood smear images. These methods were mainly built to detect malaria parasites and to run on smartphones, using two major steps, the intensity-based iterative global minimum screening (IGMS) and a customized CNN, which classifies an individual alternative based on parasitic and non-parasitic. About 1819 datasets of thick smear images were collected. The data were trained and tested using the deep learning method. The result showed an accuracy of about 93.46 + 0.32%, distinguishing both positive and negative parasitic images. The AUC shows 98.39 + 0.18%, specificity 94.33 + 1.25%, sensitivity 92.59 + 1.27% and precision 94.25 + 1.13%; whereas, the negative predictive value revealed about 92.74 + 1.09%. Therefore, Yang et al.’s method proved to have an automated detection of ground truth and image of the parasite [8].

Kassim et al. [11] also proposed a novel paradigm of the *Plasmodium* VF-Net to identify thick microscopic blood smear images to detect malaria and the patients. The research architecture was set to determine an infected malaria patient with Plasmodium falciparum or Plasmodium vivax. Therefore, the mask regional convolutional neural network (Mask R-CNN) was incorporated with Plasmodium VF-Net to detect Plasmodium parasites. Similarly, the ResNet50 classifier was used in filtering false positives. The result revealed an overall accuracy of more than 90% on both the patient level and the image [11]. Another study by Kassim et al. [18] explored the clustering-based deep-learning technique for detecting red blood cells in microscopic images of peripheral blood smears. A novel deep learning framework called RBCNet was developed for detecting and identifying red blood cells in thin blood smear pictures. This method utilized a double deep-learning architecture. The RBCNet comprises two stages: the initial step, referred to as the U-Net stage, is used in the segmented cell clusters or superpixels, and the second, described as the Faster R-CNN stage, is utilized to find small cell objects that are contained in the clusters. In their study, instead of employing region suggestions, RBCNet trains on nonoverlapping tiles and adapts to the scale of cell clusters during inference, using small memory space. This makes it suitable for recognizing minute objects or fine-scale architectural features in large images. The result revealed that RBCNet achieved an accuracy of over 97% while testing its ability to recognize red cells. Thus, the innovative double cascade RBCNet model yields significantly greater true positive and reduced false negative rates than conventional deep learning approaches [18].

In medical laboratory practice, the gold standard for malaria parasite identification and detection is a microscopic examination using a drop of a patient’s blood as a blood smear on a microscope slide. Before an examination, a blood smear is mostly stained with Giemsa stain to give parasites a distinctive appearance. A blood smear can be prepared as a thin and thick film. Parasites are more concentrated than in an equal area of a thin smear. Thick smears consist of a thick layer of red blood cells, and these are the cells lysed by the malaria parasite forming a ring, elongated, or crescent shape. The more the volume of red cells in the thick smears, the more the probability of red cells being lysed in the presence of malaria parasites. In contrast, thin smears have a lower concentration of red cells because of the smear spread. This results in a lower concentration of red cells to lysed red cell concentration. Thus, thick smears allow more efficient detection of parasites. However, thin smears are known to allow for malaria parasite species to be identified.

Identifying the most appropriate peripheral blood smear is important for improved accuracy, rapid smear preparation, and rapid diagnosis of patients, especially in regions where malaria is endemic. Even though previous studies have successfully implemented deep-learning approaches to accurately classify microscopic images of a thin or thick peripheral blood smear into infected and uninfected, none has reported the most appropriate among the two. This study aims to develop a deep learning framework capable of automating and accurately classifying microscopic images into infected and uninfected using thin and thick peripheral blood smears. Furthermore, the study aims to report which of the two peripheral blood smears is the most appropriate for use in accurately detecting malaria parasites in peripheral blood smears. Finally, we aim to evaluate the performance of our proposed model with commonly used transfer learning models.

## 2. Materials and Methods

### 2.1. Data

**Figure 2:**
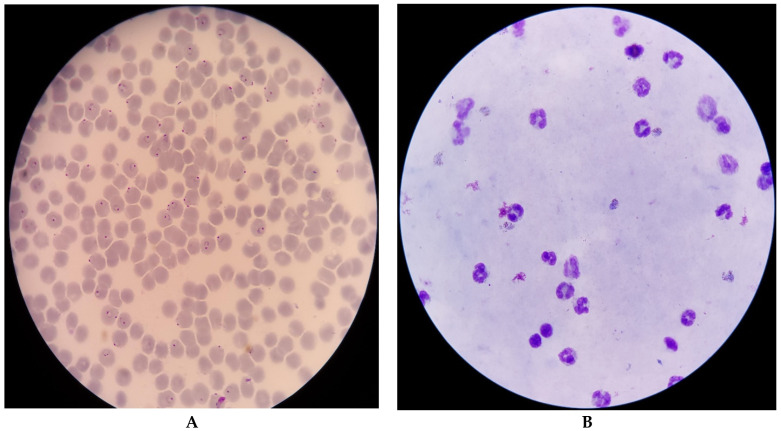
Microscopic images of thin (**A**) and thick smears (**B**) [19].

The data for this study were obtained from the National Library of Medicine and the National Institute of Health [19]. The datasets are microscopic images of thin and thick peripheral blood smears, as shown in Figure 2. The images were manually captured using a smartphone’s advanced camera through a microscope with 100× magnification. A knowledgeable specialist manually annotated them [20]. The data consist of 150 thick smears of *P. Falciparum* and *P. Vivax* and 50 thick smears of uninfected patients, as shown in Table 1. Additionally, there are 148 thin smears of *P. Falciparum*, 171 thin smears of *P. Vivax*, and 45 thin smears of uninfected patients. Subsequently, the *P. Falciparum* and *P. Vivax* of the thick smears were combined as infected and the uninfected thick smears as uninfected, whereas the *P. Falciparum* and *P. Vivax* of the thin smears were combined as infected and the uninfected smears as uninfected.

### 2.2. Data Preprocessing

Data preprocessing is a crucial and common first step in any deep learning project [21,22]. It enables raw data to be adequately prepared in formats acceptable by the network. These steps include resizing image input to match the size of an image input layer, enhancing desired features, and reducing artifacts that can bias the network. In addition, augmenting training images improves model performance by forming new and different examples to train datasets. Moreover, rich and sufficient training data will improve model accuracy and performance. Furthermore, data augmentation techniques reduce operational costs by introducing transformation in the datasets. Finally, the image denoising technique was used to remove noise and restore the true image.

### 2.3. Deep Learning 

Previous studies have shown the feasibility of deep learning to aid image classification [8,11]. Our study proposed a deep learning approach from scratch to classify patients’ microscopic images of thin and thick blood smears into infected and uninfected. A CNN takes input as an image volume for an RGB image [23]. Hence, our proposed CNN model will receive an input image with a dimension of 64 × 64 × 3. The proposed model consists of four convolution layers containing a set of kernels, kernel size, and activation functions. The convolution layer is the primary building block of a CNN and serves as a feature extractor [24]. Three max-pooling layers were added, each in the second, third, and fourth convolution layers. They serve pooling operations and help calculate the maximum value in each patch of each feature map. The result is pooled feature maps that highlight the most present feature in the patch, not the average presence of a feature in the case of average pooling. Kernel size and pool size of 3 × 3 and 2 × 2 were used across all convolution and pooling layers, respectively. These sizes are used for dimensionality reduction to reduce the number of channels in just three and two pixels of feature maps, respectively. After flattening, two dense layers with units 64 and 1 were provided. This helps in classifying images based on the output from convolution layers. It is necessary to provide our model with the ability to fit the result better and improve accuracy. As a result, the rectified linear unit (ReLu) activation function was used across all the convolution layers. The ReLu activation function helps to overcome the vanishing gradient problem, allowing the model to learn faster and perform better [25]. Nonetheless, the sigmoid activation function was adopted in the final dense layer to allow for the probabilistic prediction of output between the range 0 and 1.

Finally, we compiled our proposed model using the Adam optimizer, binary cross-entropy loss, and accuracy metrics. Our choice of optimizer was because the Adam optimizer works by combining the best properties of the AdaGrad and RMSProp algorithms to provide and optimize an algorithm that can handle sparse gradients on a noisy dataset. Similarly, we adopted the binary cross entropy loss because of the binary nature of the classification problem our study presented.

### 2.4. Transfer Learning

#### 2.4.1. VGG16

The VGG16 is one of the most popular pretrained models for image classification. First presented at the landmark ILSVRC 2014 conference, it has since established itself as the industry standard. VGG16, which the visual graphic group developed at Oxford University, outperformed the previous gold standard, AlexNet, and was soon embraced by both academia and industry for use in image classification. There are 13 convolution layers, 5 pooling layers, and 3 dense layers in the VGG16 architecture. The model receives an image of input dimension 224 × 224 × 3 with the convolution layer in 64, 128, 256, and 512 filters, respectively. The fully connected dense layer has 4096 nodes, each generating 1000 channels for 1000 classes [26].

#### 2.4.2. ResNet50

As a member of the ResNet family, ResNet50 is not the genesis model. Residual Net, the original model, was created in 2015 and was a significant step forward in computer vision. ResNet’s primary innovation is that it permits a deeper network to be constructed, thus mitigating the risk of subpar performance. The problem of vanishing gradients made training very deep neural networks challenging prior to the development of ResNet. Multiple iterations versions of the ResNet model exist, the most recent being ResNet152, which consists of 152 layers. However, transfer learning frequently begins with ResNet50, a scaled-down version of ResNet152. Five convolution and identity blocks make up the ResNet50 CNN model. There are three convolution layers in the convolution blocks and three in the identity blocks. More than 23 million adjustable parameters allow for customization of the ResNet50 [27].

#### 2.4.3. InceptionV3

InceptionV3, also called GoogleNet, is CNN architecture from the Inception family that makes several improvements, including label smoothing, factorized 7 × 7 convolutions, and an auxiliary classifier to propagate label information to lower the network. The InceptionV3 is a superior version of the InceptionV1, which was introduced as GoogleNet in 2014. It has 42 layers and a lower error rate than its predecessors. The InceptionV3 architecture consists of factorized convolutions, smaller convolutions, asymmetric convolutions, auxiliary classifiers, and grid size reduction [28].

### 2.5. Evaluation Metrics

#### 2.5.1. Accuracy

Accuracy is a metric that generally describes how the model performs across all classes. It is useful when all classes are of equal importance [29]. It is calculated as the ratio of correct predictions to the total number of predictions. Accuracy is calculated using the following:

$$Accuracy=\frac{\mathrm{True}\;\mathrm{Positive}+\mathrm{True}\;\mathrm{Negative}}{\mathrm{True}\;\mathrm{Positive}+\mathrm{True}\;\mathrm{Negative}+\mathrm{False}\;\mathrm{Positive}+\mathrm{False}\;\mathrm{Negative}}$$

#### 2.5.2. Precision

Precision reflects how reliable the model is in classifying the images as infected. The goal is to classify all the infected images as infected and not misclassify an uninfected image as infected. Precision is calculated as the ratio between the number of infected images correctly classified to the total number of infected images classified as infected (either correctly or incorrectly). Precision is calculated using the following:

$$Precision=\frac{\mathrm{True}\;\mathrm{Positive}}{\mathrm{True}\;\mathrm{Positive}+\mathrm{False}\;\mathrm{Positive}}$$

#### 2.5.3. Sensitivity

Sensitivity measures the model’s ability to detect infected samples. It only cares about how the infected images are classified. This is independent of how the uninfected images are classified. The higher the sensitivity, the more infected images are detected. Sensitivity is calculated as the ratio of infected images correctly classified as infected to the total number of infected images. It can be calculated using the following:

$$Sensitivity=\frac{\mathrm{True}\;\mathrm{Positive}}{\mathrm{True}\;\mathrm{Positive}+\mathrm{False}\;\mathrm{Negative}}$$

#### 2.5.4. F1 Score

The F1 score is an important evaluation metric in machine learning. It elegantly sums up the predictive performance of a model by combining two otherwise competing metrics-precision and sensitivity. F1 score is defined as the harmonic mean of precision and sensitivity. It is the average of precision and sensitivity and can be calculated using the:

$$F1\;score=2*\frac{\mathrm{Precision}*\mathrm{Sensitivity}}{\mathrm{Precision}+\mathrm{Sensitivity}}$$

#### 2.5.5. Confusion Matrix

**Figure 3:**
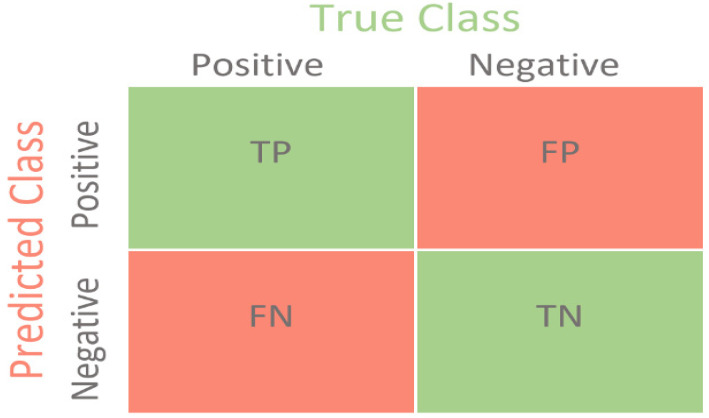
Confusion matrix [30].

The confusion matrix is used to determine the performance of the classification models for a given set of test data. It can only be determined if the true values for test data are unknown. The matrix can be easily understood, but the related terminologies may be confusing, as shown in Figure 3.

- True Negative (TN): It refers to the number of times the model correctly classifies the infected images as infected.
- True Negative (TN): It refers to the number of times the model correctly classifies the uninfected images as uninfected.
- False Positive (FP): It refers to the number of times the model incorrectly classifies the uninfected images as infected.
- False Negative (FN): It refers to the number of times the model incorrectly classifies the infected images as uninfected.

### 2.6. Model Training and Validation 

The datasets were split into 70% training and 30% test set in training the proposed model. A subset of the training set (25%) was used as the validation set. A batch size of 32 was used across the training, validation, and test set. This is aimed at controlling the accuracy of the estimate of the error gradient when training the neural network. Both the training and validation dataset were shuffled to help prevent overfitting and to ensure that batches are more representative of the entire dataset. Finally, we trained the model using 50 epochs and implemented call back and early stopping while using validation loss as a monitor. There is no rule of thumb on the number of epochs to use. It is at the discretion of the machine learning expert or researcher. We used early stopping as a form of regularization to avoid overfitting during training.

## 3. Results and Discussion

All the techniques and processes implemented in the study were carried out in the Jupyter notebook environment. Table 2 indicates the hardware and software implementation of the study. The result obtained from our study shows the relevance and capability of deep learning frameworks to be useful in classifying and distinguishing the infected microscopic image of the malaria parasite from uninfected ones. The infected datasets are microscopic images of confirmed Plasmodium falciparum (*P. falciparum*) and Plasmodium vivax (*P. vivax*), whereas the uninfected images comprise no parasites.

First, the proposed model was trained, validated, and tested using infected and uninfected images from thin smears. Then, the same process was carried out using thick smears. The result obtained, as shown in Table 3, shows the performance evaluation metrics of the model when the microscopic images of both thin and thick smears were evaluated. The proposed model’s accuracy significantly indicates the proposed model’s performance in accurately classifying infected and uninfected images using both thin and thick blood smears. However, with an accuracy of 96.97%, the proposed model performs significantly better identifying the two classes when microscopic images of thick blood smears are used. Because greater accuracy does not indicate optimum model performance [29], it is necessary to evaluate the model’s ability to classify infected images as infected and not misclassify uninfected as infected. Further, the harmonic mean of precision and sensitivity is vital as a metric to measure model performance. The sensitivity weighted average of 97.00% further indicates the improved ability of the proposed model to detect infected images when thick smears were used. The higher the sensitivity, the more an infected image is detected and classified, independent of the uninfected images detected.

**Figure 4:**
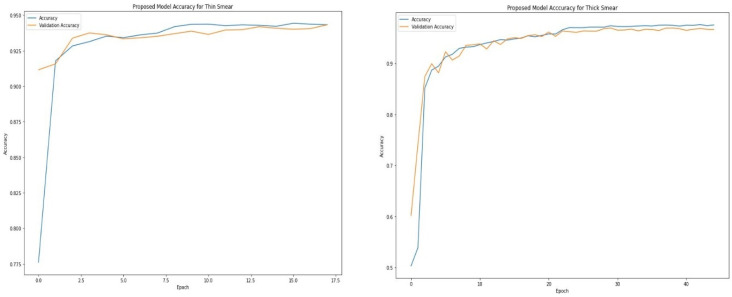
Proposed training and validation accuracy for thin and thick smears.

**Figure 5:**
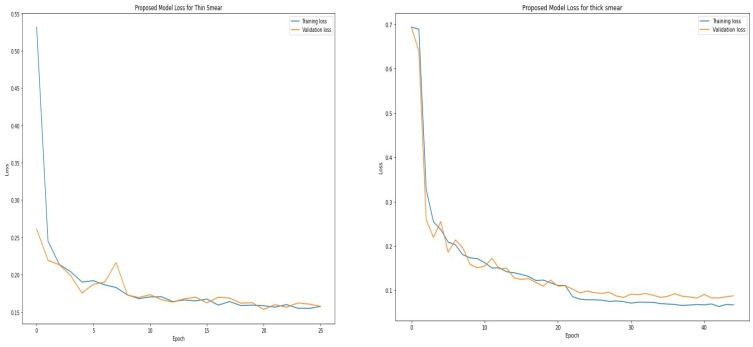
Training and validation loss for thin and thick smears.

Table 4 and Table 5 show the epoch, accuracy, and loss through the training process for thin and thick smears. As expected, the accuracy increases with an increase in the epoch, whereas the loss decreases with an increase in the epoch. However, the model eventually reaches a point where increasing epochs will not improve accuracy. The proposed model attained optimal accuracy with no significant improvement from about 16 epochs when thin smears were used and 30 epochs when thick smears were used. It is vital to monitor how the learning process is going. An ideal deep-learning model should learn useful information from the training data (generalization) at a reasonable rate. The proposed model learned useful information from the training set and validated the learned information using the unseen validation set. Figure 4 shows that the accuracy obtained during training is close to those achieved during validation. This is visible when thin and thick smears are used. Furthermore, the loss metric was used to assess model performance. Loss quantifies the error produced by the model and can be displayed in a plot commonly referred to as a learning curve. A high loss value usually means the model has erroneous output, whereas a low loss value indicates fewer errors in the model. As shown in Figure 5, the training loss indicates how the proposed model fits the training and validation data. The training and validation loss are visualized on the graph to diagnose the model performance and identify which aspect needs tuning. The proposed model produced a good fit as the training loss and validation loss decreased and stabilized at a specific point for both thin and thick smears. However, a loss of 0.07581 was produced when thick smears were used indicates better performance than 0.12348, when thin smears were used.

**Figure 6:**
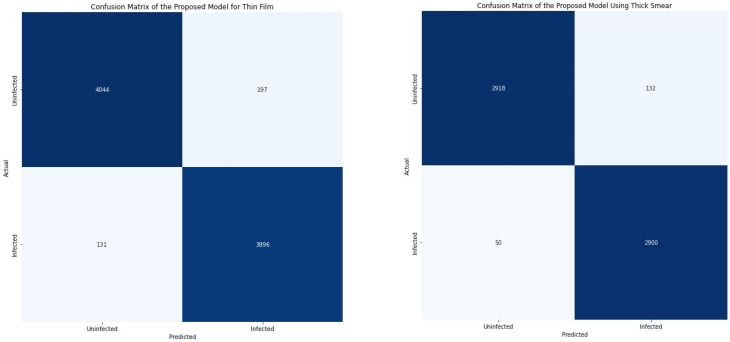
Confusion matrix for thin and thick smears.

As indicated in Figure 6, when thin smears were used, the proposed model classified 4044 microscopic images as uninfected (TP) and 3896 as infected (TN). Subsequently, when thick smears were used, 2918 images were classified as uninfected (TP) and 2900 as infected (TN). This indicates that out of the total testing image of 8268, 7940 images (96.03%) were correctly classified when thin smears were used. Furthermore, out of the total 6000 test images, 5818 (96.97%) microscopic images were correctly classified when thick smears were used. This further shows that the model performed slightly better when using thick smears. However, 328 images (3.97%) were wrongly classified when thin smears were used. In contrast, 328 images (3.03%) were misclassified when thick smears were used.

**Figure 7:**
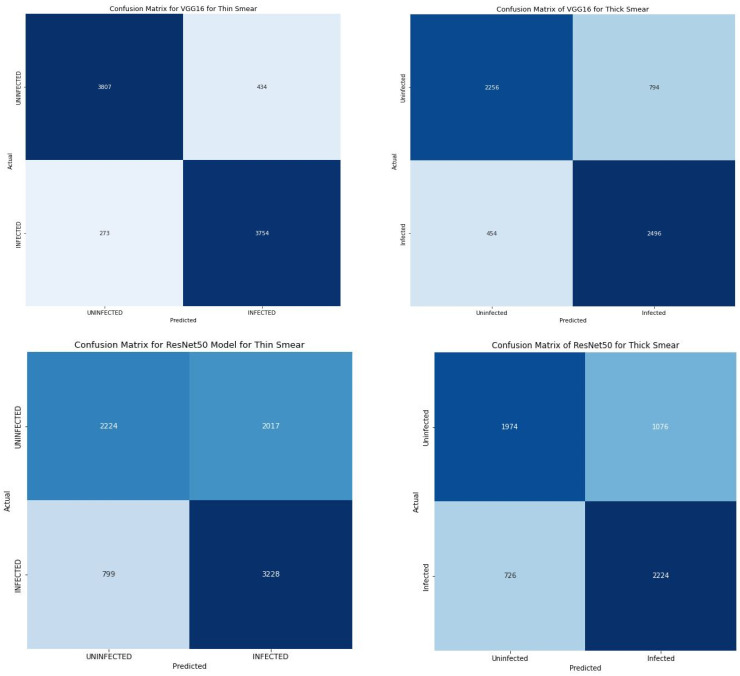

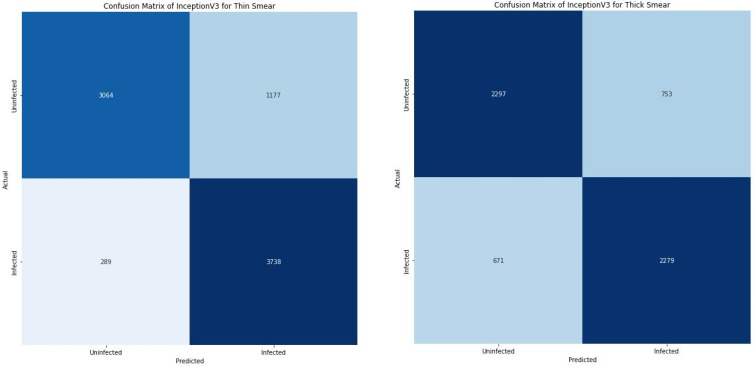
Confusion matrix of all transfer learning models.

When compared with several state-of-the art pre-trained models as shown in Table 6, it is clear that our proposed model significantly outperformed them. Further, the loss of 0.12 and 0.08 produced by our proposed model indicate a substantially low error rate. The ResNet50 model correlate with improved performance when thick smears were used. Similarly, the loss obtained from the ResNet50 model showed a decreased loss when thick smears were used. However, the performance of the VGG16 and InceptionV3 model took a different dimension. Both models produced a significant improvement in performance when thin smears were used. The VGG16 produced an accuracy and loss of 91.45% and 0.21 when thin smears were used when compared with an accuracy and loss of 79.20% and 0.45 produced when a thick smear was used. The InceptionV3 produced a similar outcome with an accuracy and loss of 82.27% and 0.39 when thick smears were used and 76.27% and 0.48 when a thin smear was used. Furthermore, the VGG16 correctly classified 3807 images as uninfected (TP) and 3754 images as infected (TN) and misclassified about 707 images (FP + FN) of the total images as shown in Figure 7.

## 4. Conclusions

This study proposed a deep learning framework to automate malaria parasite detection in thin and thick peripheral blood smears. The proposed CN model employs image augmentation, regularization, shuffling, callbacks, and early stopping. The study’s finding highlights the capability of the deep learning framework to be used for identification and classifying infected and uninfected smears using microscopic images. Our proposed model produced an accuracy of 96.97% when thick smears were used and 96.03% when thin smears were used. This performance is correlated with other performance evaluation metrics, including precision, sensitivity, recall, and F1 score. Finally, the outcome of the study indicates that regardless of the smear used, the deep learning framework produces an outstanding performance when a thin and thick peripheral blood smear are used.

## Figures and Tables

**Table 1:** Data distribution of patients.

	Thin Smear	Thick Smear
*P. falciparum* patients	148	150
*P. vivax* patients	171	150
Uninfected patients	45	50

**Table 2:** Hardware, software, and libraries Implementation.

	Hardware/Software/Libraries	Setting
1	Windows	Windows 10 Pro
2	Random access memory (RAM)	64.0 GB
3	Graphics processing unit (GPU)	NVIDIA GeForce RTX 3070
4	Operating system	64 bit operating system x64-based processor.
5	Processor	11th Gen Intel (R) Core (TM) i7-11700KF @3.60GHz 3.60 GHz.
6	Storage Space:	1 TB*1
7	Programming language	Python
8	Frameworks/Libraries	TensorFlow, Keras, NumPy, Pandas, Pathlib, matplotlib, seaborn, and SkLearn

**Table 3:** Evaluation metrics for proposed model.

	Precision %		Sensitivity %		F1 Score %		Accuracy %	
	Thin Smear	Thick Smear	Thin Smear	Thick Smear	Thin Smear	Thick Smear	Thin Smear	Thick Smear
Infected	95.00%	96.00%	97.00%	98.00%	96.00%	97.00%	96.03%	96.97%
Uninfected	97.00%	98.00%	95.00%	96.00%	96.00%	97.00%		
Weighted Average	96.00%	97.00%	96.00%	97.00%	96.00%	97.00%		

**Table 4:** Training epoch, accuracy, and loss for thin smear.

Epoch	Accuracy	Loss
1	82.19%	0.3945
6	93.49%	0.1958
11	94.01%	0.1714
16	94.43%	0.1609
21	94.28%	0.1570
26	94.50%	0.1506

**Table 5:** Training epoch, accuracy, and loss for thick smear.

Epoch	Accuracy	Loss
1	50.30%	0.6936
5	89.46%	0.2372
10	93.25%	0.1714
15	94.55%	0.1397
20	95.47%	0.1171
25	96.98%	0.0788
30	97.37%	0.0746
35	97.38%	0.0702
40	97.31%	0.0702
45	97.54%	0.0673

**Table 6:** Performance Evaluation of all models.

	Class	Precision %		Sensitivity %		F1 Score %		TP		FP		FN		TN		Accuracy %		Loss	
		Thin	Thick	Thin	Thick	Thin	Thick	Thin	Thick	Thin	Thick	Thin	Thick	Thin	Thick	Thin	Thick	Thin	Thick
Proposed Model	Inf.	95.00	96.00	97.00	98.00	96.00	97.00	4044	2918	197	132	131	50	3896	2900	96.03	96.97	0.12	0.08
	Uninf.	97.00	98.00	95.00	96.00	96.00	97.00												
VGG16	Inf.	76.00	90.00	85.00	93.00	80.00	91.00	3807	2256	434	794	273	454	3754	2496	91.45	79.20	0.21	0.45
	Uninf.	83.00	93.00	74.00	90.00	78.00	92.00												
ResNet50	Inf.	62.00	67.00	80.00	75.00	70.00	71.00	2224	1974	2017	1076	799	726	3228	2224	65.94	69.97	0.62	0.57
	Uninf.	74.00	73.00	52.00	65.00	61.00	69.00												
InceptionV3	Inf.	75.00	76.00	77.00	93.00	76.00	84.00	3064	2297	1177	753	289	671	3738	2279	82.27	76.27	0.39	0.48
	Uninf.	77.00	91.00	75.00	72.00	76.00	81.00												

## Data Availability

Data will be provided upon request.
